# Continuous and Long-Term Volume Measurements with a Commercial Coulter Counter

**DOI:** 10.1371/journal.pone.0029866

**Published:** 2012-01-17

**Authors:** Andrea K. Bryan, Alex Engler, Amneet Gulati, Scott R. Manalis

**Affiliations:** 1 Department of Biological Engineering, Massachusetts Institute of Technology, Cambridge, Massachusetts, United States of America; 2 David H. Koch Institute for Integrative Cancer Research, Massachusetts Institute of Technology, Cambridge, Massachusetts, United States of America; Texas A&M University, United States of America

## Abstract

We demonstrate a method to enhance the time resolution of a commercial Coulter counter and enable continuous and long-term cell size measurements for growth rate analyses essential to understanding basic cellular processes, such as cell size regulation and cell cycle progression. Our simple modifications to a commercial Coulter counter create controllable cell culture conditions within the sample compartment and combine temperature control with necessary adaptations to achieve measurement stability over several hours. We also wrote custom software, detailed here, to analyze instrument data files collected by either this continuous method or standard, periodic sampling. We use the continuous method to measure the growth rate of yeast in G1 during a prolonged arrest and, in different samples, the dependency of growth rate on cell size and cell cycle position in arrested and proliferating cells. We also quantify with high time resolution the response of mouse lymphoblast cell culture to drug treatment. This method provides a technique for continuous measurement of cell size that is applicable to a large variety of cell types and greatly expands the set of analysis tools available for the Coulter counter.

## Introduction

Cell size is a fundamental property of all organisms and tissues. Size is coupled to cell cycle progression and affected by both internal and external cues, as well as certain disease states. The measurement of cell size over time offers insight into the rate at which cells translate energy derived from nutrients into cellular biomass, and this information can be applied to molecular-level knowledge to further understanding of cell size regulation and predict cell fate. Size measurements by single cell tracking provide the highest level of detail, but are low throughput and face technical challenges because cells move or drift and require a steady nutrient supply [1]–[3]. Population-scale measurements at fixed time intervals evaluate a large number of cells, but are often collapsed into qualitative descriptions or a single data point, such as a change in population mode or average [4], [5]. Moreover, population-scale data frequently lack the time resolution necessary to quantify any fast kinetics during a culture's response. A large-scale *continuous* size measurement captures with high time resolution valuable statistics about the population's size heterogeneity, describes how the average cell of any given size behaves, and more precisely identifies when a population responds to environmental perturbations.

Continuous population-scale volume measurements have not been achieved, mainly due to the lack of instruments and analysis tools. In addition to the requirement that cells be kept in culture conditions for the entirety of the timecourse, this style measurement must be ultra-high throughput without sacrificing precision. Tools for measuring cell volume are mostly limited to image analysis, light scatter, and the resistive-pulse (Coulter) technique. Image analysis enables relatively high resolution in a focused horizontal plane, but non-spherical cells larger than the objective's depth of field necessitate z-stack imaging and a computationally slow reconstruction process [6], [7]. Image acquisition may be as fast as 30 cells per second if cells are imaged in parallel, but the necessary processing to calculate volume can be slow and constitutes a major source of error. Forward scatter (FSC) measurements can achieve rates exceeding 10 000 cells per second, but FSC is more closely related to cross-sectional area than volume, and it assumes all cells are spherical and have identical optical properties [8], [9]. Deviations in cell shape and content introduce error to FSC measurements and this error has been reported as instrument-dependent [10], which makes it difficult to compare results across studies.

**Figure 1:**
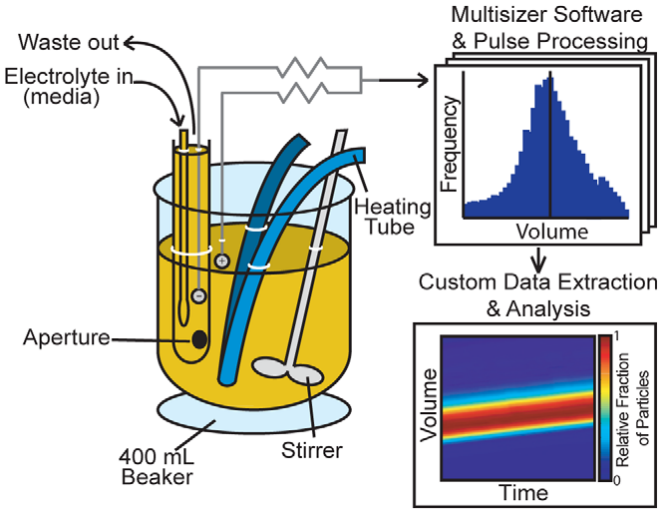
Schematic of setup within the sample compartment of a Beckman-Coulter Multisizer 4. The instrument electrolyte is exchanged for cell media and 400 mL of cell culture is in a beaker on the sample platform. A heating tube and the Multisizer stirrer maintain culture temperature and a homogeneous suspension. The heating tube is connected to a temperature-controlled recirculating waterbath filled with deionized water, which minimizes electrical interference. Each 150 s file is stored by the Multisizer software and custom software reads the single-particle data from multiple files to plot volume for the timecourse.

The commercial Coulter counter is also high-speed (∼2 000 cells per second) but, in contrast to FSC, its output is directly proportional to cell volume. The Coulter principle states that a cell transiting an aperture decreases the aperture's electrical conductivity in proportion to the volume of the cell [11]. The commercial instrument's aperture is on a test tube-like structure that is directly immersed in a sample beaker (Figure 1), and cells are driven via negative pressure from the beaker into the tube by way of the aperture. The commercial version is designed for “instantaneous” volume profiling of large cell populations at discrete time points; however, many biological studies require dynamic measurements over an extended timecourse with quantitative analysis of how cells change with time. To address this, we present modifications and analysis tools for a commercial Coulter counter to continuously acquire population data from active cell culture and quantitatively describe cell response as a function of both volume and time.

## Results and Discussion

### Instrument modifications for measurement of culture volume response

The basic requirement for a large-scale continuous cell volume measurement is the ability to maintain cell cultures in temperature-controlled media and homogeneous suspension during sampling. If the Coulter counter's sample beaker is filled with cell medium and the electrolyte inside the aperture tube is commercial solution (Isoton II), there is a gradient of electrical conductivity through the aperture sensing zone that causes the initial volume measurements to be unreliable (Figure S1). We found volume measurements in media are stable only when the instrument electrolyte closely matches that of the sample. Thus, we replaced the Isoton contents of the instrument with 0.2 µm filtered cell medium matched to each culture protocol (Figure 1). Additionally, we used the Multisizer 4 accessory stirrer to maintain a homogeneous cell suspension. In order to achieve temperature-controlled conditions within the commercial instrument, we passed into the sample compartment polyurethane tubing connected to a temperature-controlled recirculating bath (Figure 1). The recirculating bath must be filled with nonconductive liquid (*e.g.*, deionized water, oil) to shield the instrument from catastrophic electrical noise in the external environment. Even weak conductive paths between the environment and sample compartment interfere with the Coulter counter's electrical measurement. Although the ideal method for controlling culture temperature is a fluid-jacketed beaker and temperature feedback with a recirculating bath, we observed temperature stability to be within 1°C using our system (Figure 1). Temperature sensing methods should not electrically connect the external environment to the sample compartment, in the same way that fluid paths for temperature control must not interfere with the measurement. Temperature may be monitored either between file recordings with an external thermocouple or continuously by a thermocouple entirely contained within the sample compartment.

### Commercial software set-up and description of custom data processing

In addition to establishing cell culture conditions, there are critical settings in the Multisizer software that allow for stable long-term measurements. One limitation of the commercial software is the 525 000 cell limit for each data file written. By recording a new data file every 150 s interspersed with aperture tube flushes, millions of single-cell measurements for a culture may be collected over several hours. Regular flushing of the aperture tube reduces continuity (data are collected during ∼75% of the measurement), but ultimately results in higher quality data. By this method, a typical two-hour timecourse produces several million volume measurements distributed across ∼40 raw Multisizer data files that are batch-processed by our custom software. Details for the extraction of time data from Multisizer files (*.#m3 or *.#m4, model-dependent) are provided in the Materials and Methods, and MATLAB (MathWorks, Natick, MA) code is provided in File S1. The fundamentals of the volume extraction are as follows:

1. Cell diameter is calculated from each peak height, according to Beckman-Coulter, as:
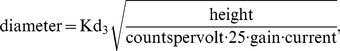
(1)where Kd, gain, and current are constants reported in the header of each file. Height is the sum of a correction factor and the first of five hexadecimal-encoded values recorded for each cell measurement. Countspervolt is 838 870 V^−1^.
2. Particle volume is calculated as π/6*diameter^3^.

The Coulter principle measures volume, but the traditional output parameter for the commercial instrument is the spherical equivalent diameter, or Heywood diameter, given by Equation 1. Errors in the volume measurement result from elongated particles that widen the size distribution as a result of the instrument's orientation-dependent measurement [12]–[14] and particles that travel near the outer edges of the aperture [15]. Since particle path is related to pulse width, or time the particle spends in the aperture, pulse width could be used to exclude many of these erroneous measurements. Multiple particles within the aperture are also a source of measurement error and increased pulse width.

### Assessment of error during long-term volume measurements

**Figure 2:**
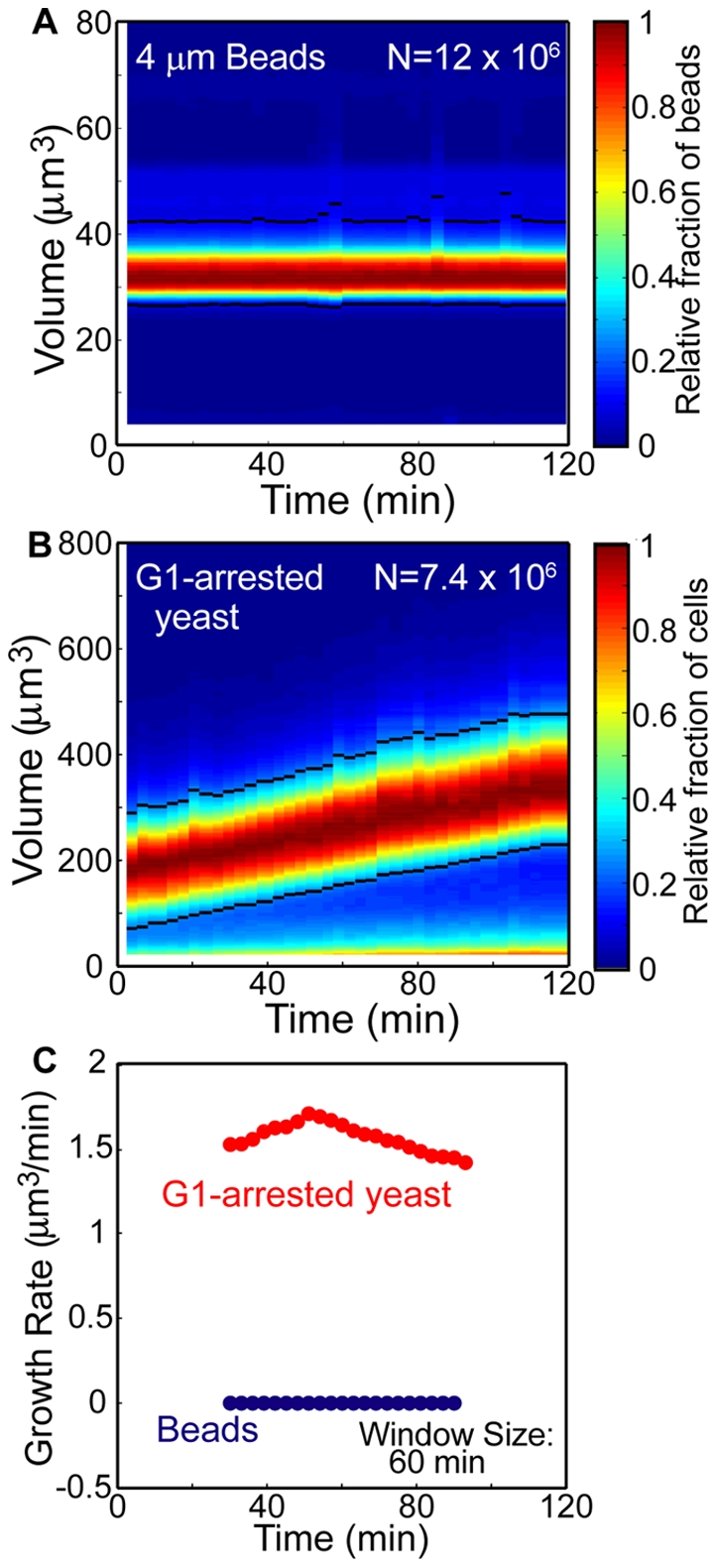
High resolution volume timecourse and growth rates of particles and cells. (**A**) 4.000±0.033 µm diameter beads (Duke Scientific) and (**B**) *cdc28-4* G1-arrested yeast volume measurements over a 2 hour timecourse. Color designates the relative fraction of particles in each 150 s measurement with the indicated volume (colorbar at right). Black lines designate the exclusion bounds used for growth rate calculations. (**C**) Growth rates for bead (blue, **A**) and yeast (red, **B**) data calculated by linear regression on a 60 min window shifted every 3 min. Bead data growth rates are 0.004±0.037 µm^3^/min (mean ± SD) and provide an estimate of measurement error. Additional error estimates were performed on 7.979±0.075 µm diameter beads (Duke Scientific) (growth rates: 0.013±0.169 µm^3^/min) and formaldehyde-fixed cells (growth rates: −0.022±0.118 µm^3^/min). Continuous sampling and single particle data increases statistical significance for growth rate data and makes possible the analysis of growth rates with different averaging windows for correlation with specific biological events.

In order to investigate the stability of continuous and long-term data acquisition, we measured beads and G1-arrested yeast (*cdc28-4*, Table 1) over a 2-hour timecourse (Figure 2AB). These data are represented by a colormap in which the number of cells located in a small area is designated by a color. Any deviation in the bead volume distribution is entirely due to measurement error. G1-arrested yeast data provide an example of a biological sample actively maintained and measured within the instrument. The G1 arrest is achieved by a temperature-sensitive allele of Cdc28, a protein kinase in yeast required for passage through Start [16]. At high temperature (>34°C) the protein is inactivated and cells arrest in G1. Colormaps of volume timecourses are drawn directly from single-particle data rather than exported histograms and may be constructed with any time and volume resolution. Occasionally, debris partially occludes the aperture and introduces an error that is resolved by a flush or unblock of the aperture tube and the subsequent start of a new file (Figure S2). The commercial software does not save data if there is a flush or unblock before the end of the file and the number of automatic unblocks is limited by the commercial software to 9. Data skewed by debris were identified as those files in which a specific feature of the size distribution was greater than that of the two neighboring files (further details in Materials and Methods), and these were replaced by data interpolated from the neighboring files. This interpolation criterion removes short timescale fluctuations (∼150 s), which are not biologically relevant for the samples we measured. Faster events may require alternative constraints. Volume timecourse colormaps without interpolation are provided in Figure S3AB for comparison. As an alternative to data interpolation, spiking each cell sample with a large and small internal bead standard could be used to correct data during partial occlusions by imposing the constraint that bead volume distributions do not change during the experiment's timecourse. Ideally, a weak and rapid pressure reversal that does not interrupt data collection would eliminate the need for interpolation.

**Table 1:** Yeast strains used in this study.

Strain	Relevant genotype
A4370	*MATa, cdc28-as1 (w303)*
A17132	*MATa, cdc28-4 (w303)*
A702	(WT) MATa/MATalpha, ade2-1/ade2-1, leu2-3/leu2-3, ura3/ura3, trp1-1/trp1-1, his3-11,15/his3-11,15, can1-100/can1-100 *(w303)*

Continuous data collection enables growth rates to be reported with high frequency and nearly any averaging or smoothing window size. The calculated growth rates for beads (Figure 2C, blue) establish the method's growth rate resolution to be as low as 0.111 µm^3^/min (three standard deviations, 60 minute averaging window) and significantly smaller than the growth rates observed for G1 arrested cells (Figure 2C, red). The arrested cell sample's growth rate remains elevated (Figure 2C), which is supported by previous results in which G1-arrested cells maintain growth over several hours [4]. As the averaging window sizes increases, the calculated rates converge to the average. For some samples, smaller averaging windows may reveal features that correlate with specific biological events.

### Rapid detection of growth rate perturbations in mammalian cell culture

**Figure 3:**
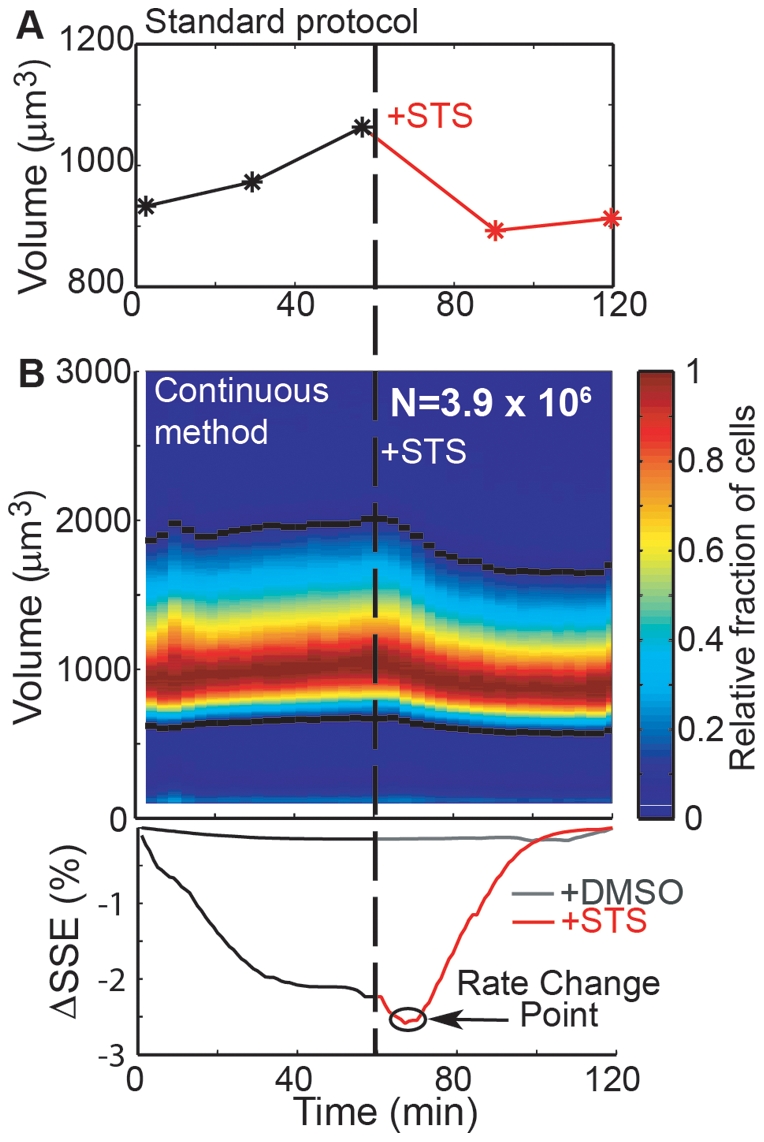
Timing of drug response by continuous volume data. After exposure to 0.5 µM staurosporine (STS), mouse lymphoblast leukemia cells (L1210) exhibit an average volume decrease, likely associated with the early stages of apoptosis. (**A**) In a standard protocol, volume measurements are recorded and a histogram mode is reported (stars) every 30 minutes. With these data, the time at which cells respond to environmental perturbations can be determined with ∼30 minute precision. (**B**) Continuous volume data for the same sample (colormap) provides a more complete description of the treatment's effect. For example, not only does a volume decrease occur following STS treatment, but there is a decrease in the population's variation. These data are then quantitatively analyzed to determine the time at which the measurement detects the culture response. Black lines designate the exclusion bounds (**B**, colormap black lines), or data removed from analysis. A linear regression is calculated for data before and after a breakpoint varied across the entire timecourse. For each of these points, the goodness of fit is measured by the sum of squared errors (SSE) and the minimum SSE indicates the time at which a change in volume growth rate is detected—the rate change point. L1210 cells treated with equal volume DMSO exhibit no significant rate change point, and a response in staurosporine-treated cells was detected at ∼9.7 min after drug exposure.

In order to determine the time resolution at which cell response may be detected and demonstrate the method for mammalian cells, we continuously measured the volume of a mouse lymphoblast leukemia (L1210) cell line before and after treatment with staurosporine. Staurosporine is an inducer of the intrinsic apoptotic pathway, for which the first stage is apoptotic volume decrease (AVD) [17]. AVD is clearly detected within 30 minutes by standard protocols that track the mode of a volume distribution measured at fixed time intervals (Figure 3A), but a general and quantitative method for determining how early this change occurs could provide insight into the time required by certain pathways to induce changes to a cell's biophysical properties, such as size. During the initial stages of AVD, ion pumps that maintain homeostatic ion balance across the cell membrane shut down [17], [18], thus changes to cell size in response to staurosporine are similar to those during osmotic shock. The time for a change in cell size to occur consists of the time needed for the cells to respond and the system to detect the change. In order to determine this time, L1210 cells were measured for 1 hour, treated with 0.5 µM staurosporine, and measured for an additional hour (Figure 3B, upper panel). These data were analyzed with a bilinear model (similar to [19]) in which the entire dataset is broken in two and a linear regression is performed on each part. The location of the separation is varied across the timecourse and the goodness of fit was measured by the sum of squared errors from both regressions. A decrease in the sum of squared errors indicates an improvement in the bilinear fit (Figure 3B, lower panel). A mock-treated sample (equal volume DMSO) demonstrates no improvement in the fit because its measured volume is constant and there is no optimal point for the separation. Cells treated with staurosporine show a dramatic fit improvement at ∼9.7 min. This analysis highlights the continuous method's ability to precisely identify a population's response to changes in environmental conditions without prior knowledge of the timing or timescale of this change. The brevity in the cells' time to respond indicates that the size changes must occur by fast-acting pathways, such as those reported in previous literature.

### Growth rate calculations for size-based subpopulations

**Figure 4:**
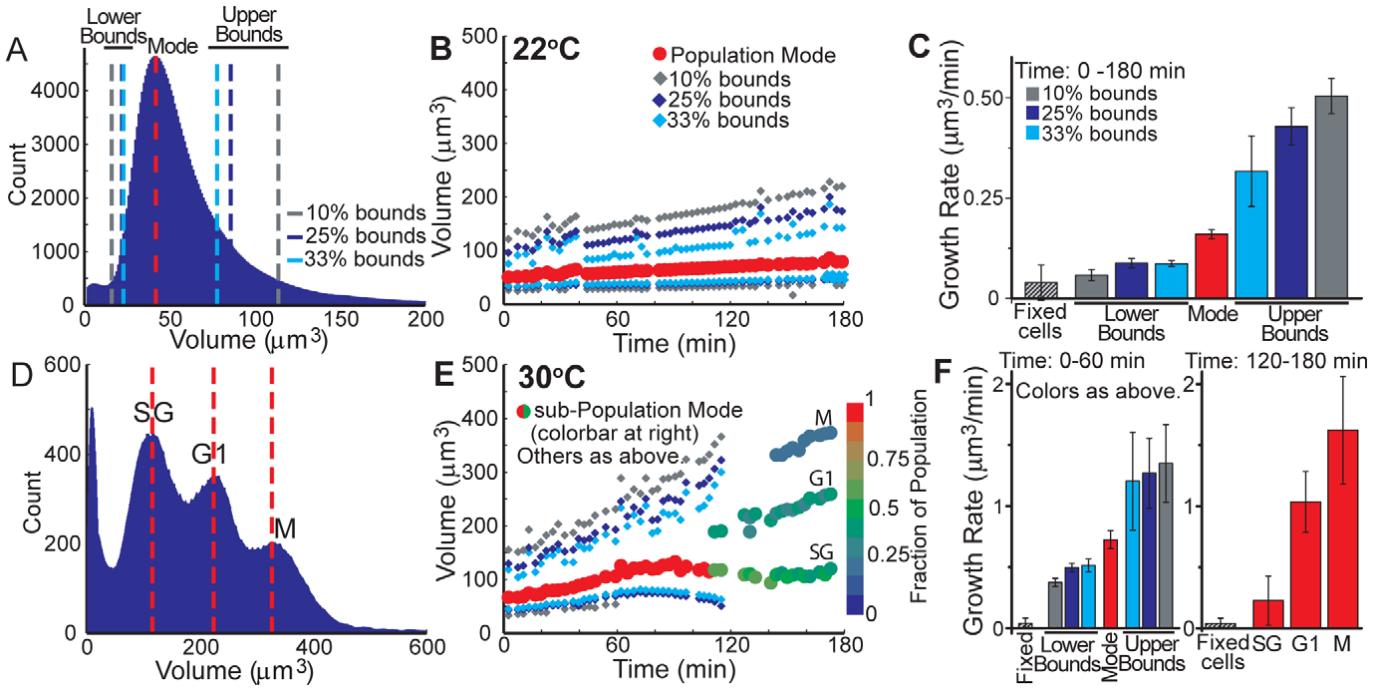
Subpopulation tracking by volume histograms. Continuous volume measurements of an arrest in *cdc28-as1* yeast over a 3-hour timecourse show cells arrested at various points in the cell cycle. (**A**) Histogram features, such as bounds, on single-mode volume distributions separately analyze large and small cells. (**B**) At room temperature (∼22°C), cell volume steadily increases with a single population mode, and (**C**) the arrested cells' volume growth rates increase with cell size. Error bars on *cdc28-as1* data indicate the 95% confidence interval for the linear regression. The growth rate average (n = 6) and measured standard deviation (error bar) for formaldehyde-fixed cells with a 60 min window are shown for comparison to measurement error. (**D**) Subpopulations emerge at the optimal temperature for yeast (∼30°C) and a later timepoint (∼120 min), and the mode of each subpopulation is identified and tracked. These subpopulations likely represent slow-growing (SG), G1-arrested (G1), and metaphase-arrested (M) cells. (**E**) Histogram features are determined for each 150 s file, similar to those in **B**. The color of the solid circles indicates the fraction of cells in each subpopulation. Growth rates calculated with data in **B** and **E** are by linear regression on a 60 or 180 min window, as indicated. (**F**) Growth rates are separately calculated for a one hour window before and after the emergence of subpopulations. Fixed cell data is identical to that in **C**. As observed for room temperature measurements, growth rates are related to cell size and likely a result of the multi-phase arrest.

We next used this method to track populations of cells other than those represented by the mode of a histogram. We first investigated yeast growing at room temperature and arrested by a kinase inhibitor (1-NM-PP1) that inactivates Cdc28 (*cdc28-as1*, Table 1). For single-peaked histograms, which we observed for timecourses measured at room temperature, small and large cells may be separately tracked by specific histogram features, such as bound pairs. Bound pairs are defined as the volume for which the count is X% of the mode (Figures 4A) and consist of one bound on each side of the mode. Bound pairs ease the tracking of changes in histogram shape over large datasets (Figure 4B). Although the result is similar to the volume timecourse colormaps shown in Figures 2 and 3, this information enables growth rate data to be calculated independently for subpopulations of the culture measured together. In arrested cells and other samples in which cells do not divide, these growth rates are correlated to cells of a specific size and cell cycle phase (Figure 4C). Yeast arrested in the cell cycle have a growth rate that is dependent on cell size, which is similar to observations in cycling populations [20], but within a given size class the growth of arrested cells is relatively constant throughout the timecourse. Thus, Cdc28-inactivated yeast appear to have a diminished capacity to accelerate growth even as cells increase in size.

For samples with emerging subpopulations, the modes of each can be identified as local peaks and tracked individually (Figure 4D). We observed multi-peaked distributions to occur in cells with Cdc28 inactivation and grown at the optimal temperature for yeast (30°C). During the first hour of arrest the distribution is single-peaked and analysis is as described previously, but subpopulations later appear as a result of elevated growth rates (Figure 4EF). The G1-labeled population closely correlates with a population grown in parallel culture and maintained in an incubated shaker (30°C, 300 RPM). Although Cdc28 inactivation by 1-NM-PP1 arrests most cells in G1, a significant fraction of the population arrests as large budded cells. This is in agreement with work by Bishop, et al. [21], which reports an arrest with both unbudded G1 cells and large-budded G2/M cells at the same inhibitor concentration (5 µM). The M-labeled population likely represents the metaphase-arrested cells we observed in parallel culture. These G1- and metaphase-arrested populations are also present in the cultures grown at room temperature, but their size distributions are indistinguishable. The SG-labeled population represents a population of slow-growing cells that are not present in a flask-grown culture and are not altered by sonication. This subpopulation may be sensitive to reduced aeration in the sample compartment and result from *cdc28-as1* cells' deficient growth [21]. The proportion of cells in each subpopulation is color-coded for reference (Figure 4E). The widening of the distribution (Figure 4BE, distance between bound pairs) is a result of the size-dependent growth rates and diverging subpopulations that likely reflect cell cycle position.

**Figure 5:**
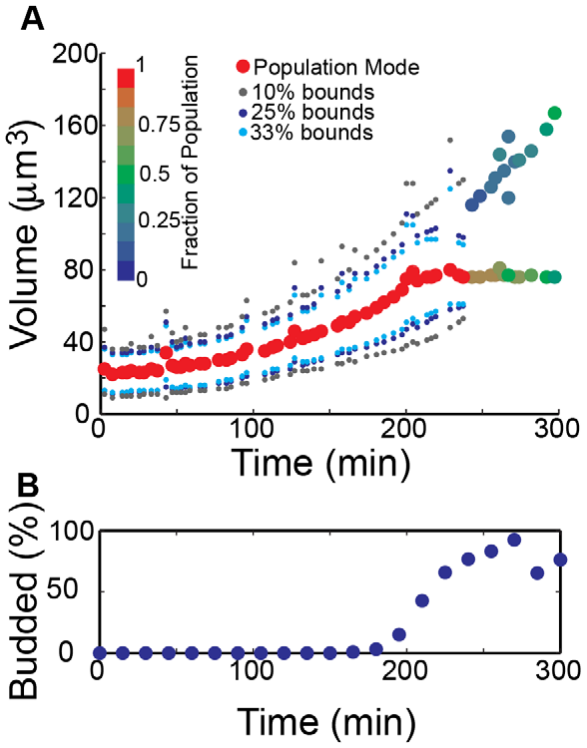
Continuous volume measurements of cell cycle-synchronized yeast. (**A**) A population of G1-phase yeast were collected via centrifugal elutriation and volume was continuously measured as cells synchronously progressed through the cell cycle. Size classes are indicated by the calculated bound pairs and the rate of growth for all size classes increases throughout the timecourse, even with the prolonged G1-phase. As the timecourse progresses, the cells lose synchrony and a bimodal population emerges. After 240 min, the mode of each subpopulation is tracked and bound pairs are omitted. (**B**) Fraction of budded cells in a flask-grown culture prepared for Figure 5A. The presence of buds indicates cell cycle position, and there is good agreement between the emergence of budded cells in the flask-grown culture and the bimodal population in cells grown within the instrument sample chamber.

We next wanted to compare the results of arrested cells to measurements for a population of synchronously proliferating cells. To accomplish this, we isolated G1-phase diploid cells (Table 1) by centrifugal elutriation and resuspended the population in YEPD for synchronous cell cycle progression. The elutriated cells (Figure 5A) have a prolonged G1-phase due to their initial small size and overnight growth in raffinose-supplemented media. As indicated by the presence of budding, these cells begin to enter S-phase near 200 min (Figure 5B). Bud calculations were recorded from a parallel flask-grown culture, and the volume distributions' modes from these aliquots closely match those of the continuously measured population (Figure S4). The G1-phase growth of elutriated cells (Figure 5A) is better fit to an exponential rather than a linear function (Table 2), which is in contrast to the more linear growth pattern of G1-arrested cells (Figure 2B and 4B). This suggests that exponential growth in proliferating yeast can be interrupted by changes to cell cycle progression. The growth rate continues to increase as cells progress through S-phase, and subpopulations emerge concurrently with the appearance of a second generation of cells in the parallel culture (Figure 5AB, 225 min). As expected, the population begins to lose synchrony over time and the calculated bounds diverge.

**Table 2:** Goodness of fit for exponential and linear growth patterns.

	10% Bounds		25% Bounds		33% Bounds		Mode
	Lower	Upper	Lower	Upper	Lower	Upper	
**R^2^ of Linear Fit**	0.938	0.896	0.943	0.896	0.943	0.903	0.932
**R^2^ of Exponential Fit**	**0.994**	**0.950**	**0.995**	**0.949**	**0.992**	**0.932**	**0.972**

Linear (y = ax+b) and exponential (y = Ae^bx^) functions were fit to the bound and mode data of Figure 5A. Analysis was limited to data between 0 and 240 min, after which the population loses synchrony and is bimodal. The goodness of fit is determined by R^2^, or the coefficient of determination, which measures scatter surrounding a fitted function. Higher R^2^ values indicate a better fit and all populations from Figure 5A are better fit by the exponential function.

### Conclusion

Analysis of changes to cell size distribution provides fundamental insight into cell size regulation. Although there are well-established cell size measurement tools, each have a fundamental tradeoff between throughput and time resolution. The method described here enhances the time resolution of a commercial Coulter counter's rapid, single-cell volume measurement by creating cell culture conditions within the sample compartment and improving long-term measurement stability. We also detail the requirements for extraction of volume and corresponding time data for timecourse analyses longer than a single file's measurement time, which is applicable to data collected by standard periodic sampling and the continuous method presented here. These methods for the extended analysis of cell size distributions are readily accessible to all users of a Beckman-Coulter Multisizer. We employ this method to describe cell growth as a function of size and cell cycle position in yeast and identify the response time for a mouse lymphoblast cell culture after drug treatment, but it may be applied to a variety of suspension cell systems and biological questions that could benefit from dynamical cell size measurements.

## Materials and Methods

### Multisizer 4 measurements

The system electrolyte was replaced with media closely matched to the cells' growth media. A 400 mL Multisizer beaker with pre-warmed growth media was equilibrated to a sample-appropriate temperature by a polyurethane heating tube connected to a temperature-controlled recirculating bath (NESLAB RTE-111) and integrated stirrer (speed for yeast: 15, L1210: 5). Cells were added to the prepared beaker for a final concentration of ∼7500 mL^−1^ (5–7% coincidence correction). Back-to-back 150 s measurements with a 100 µm aperture tube and intermediate flushes were recorded for the duration of the timecourse. Occasionally, debris remained on the aperture after a flush, and an unblock operation was initiated by the user if a sharp increase in aperture resistance occurred during the first half of a measurement. For long timecourses, the sample beaker volume was replenished with pre-warmed (if necessary, pre-treated) growth medium to maintain sample temperature and sufficient volume for measurement. Alternatively, a basic pump system contained within the sample chamber may be implemented to continuously exchange media during the measurement.

### Yeast strains, growth conditions and sample preparation

Cells were grown to OD_600_<0.7 in YEP supplemented with 2% glucose (YEPD) at room temperature (∼21°C). For *cdc28-as1*, cells were arrested with 1-NM-PP1 (5 µM) at either room temperature or 30°C, and for *cdc28-4*, cells were arrested by temperature shift (34°C). After 1 h at arrest conditions, yeast were washed via vacuum filtration, resuspended in 0.2 µm filtered YEPD, sonicated, and transferred to a measurement beaker with identical arrest conditions (*cdc28-as1*: YEPD with 5 µM 1-NM-PP1 at 21°C or 30°C, *cdc28-4*: YEPD at 34°C). The Multisizer system electrolyte was 0.2 µm-filtered YEP. Additional pre-warmed growth medium was added after 2 h of measurement to better maintain sample temperature and increase sample volume. The first hour of arrest conditions was not measured because it mostly consists of the population collecting into the arrested phase and does not accurately portray an arrested population.

### Elutriation

Cells were grown overnight in YEP +2% raffinose at 30°C, synchronized by centrifugal elutriation [22], and resuspended in YEP +2% glucose at 30°C. Aliquots from a parallel flask-grown culture were collected into 4% paraformaldehyde at indicated timepoints for quantifying the presence of buds. The Multisizer system was prepared and maintained as described above.

### L1210 growth conditions and sample preparation

Cells were grown at 37°C in L-15 medium (Gibco/Invitrogen, Carlsbad, CA) supplemented with 0.4% (w/v) glucose, 10% (v/v) fetal bovine serum, 100 I.U. penicillin, and 100 µg/mL streptomycin. Cells were passaged every 2–3 days to maintain a cell concentration of ∼100,000 mL^−1^. For measurement, cells were transferred to the Multisizer beaker with pre-warmed medium identical to growth medium and allowed to equilibrate for 30 min before measurement. After ∼1 hour of measurement, cells were treated with 0.5 µM staurosporine or an equal volume of dimethyl sulfoxide (DMSO; mock-treated control) and measured for an additional hour. The Multisizer system electrolyte was L-15 medium.

### Data extraction, interpolation, and analysis

Raw Multisizer files were batch-processed with custom MATLAB software (See File S1 and Figure S5). This software is necessary for timecourses longer than a single file because combining files within the commercial software resets the start time of each file to zero and makes rate analysis for cell cycle progression or drug response impractical. Furthermore, data exported from the commercial software are limited to 5 010 pulses per file and files must be handled individually. The custom software batch-processes raw Multisizer data files to acquire time and volume data for all measurements and extend the time axis beyond that of a single file (Figure 1). The method for volume data extraction is described in the main text. The corresponding time data are provided in two sections of the raw Multisizer file (*.#m4). Every 200 ms the instrument records the time passed (“[#TSms]”) and the number of measurements that have occurred since the measurement began ([#TSpulses]). Volume measurements are linearly distributed across each 200 ms time step such that each cell measurement has at least 200 ms time resolution.

For each file, the lowest 2% of data by volume were excluded from analysis as instrument noise. Bound pairs were determined for each file and data outside of a certain bound pair (exclusion bounds), typically the 10% bound pair, were excluded from rate analysis. Extreme size measurements skew the rate analysis and typically represent instrument noise and culture debris.

Large and instantaneous changes in the size distribution were interpreted as a partial occlusion of the instrument aperture or some other instrument error. The files identified as “debris” files contained a 25% bound pair that was greater than 1.025 times the corresponding bound pair in either of the neighboring files. These files were replaced with data interpolated from the two neighboring files and linearly spaced across the entire 150 s measurement. Interpolated data were written as a new Multisizer file and used for subsequent analysis.

Since data collected over an entire timecourse are usually too large to be imported in its entirety, files required for analysis on each averaging window are imported and cleared before analysis on subsequent averaging windows begin.

## Supporting Information

Figure S1 **Volume measurements are unreliable (up to 20% error) for mismatched electrolyte and diluent conditions.** (**A, B**) For a mismatched system electrolyte (inside aperture tube) and sample solution (beaker) there is a ∼30 s period required for the measurement to stabilize. During this period the sample solution fills the inside of the aperture tube and finally creates matched solution conditions across the aperture sensing zone. During a continuous measurement, this drift would be observed after every instrument flush, or between each recorded file (every 150 s). (**C,D**) Volume measurements are stable through the entire measurement if the system electrolyte and sample solution are identical.
(TIF)

Figure S2 **Examples of interference.** In general, interference is identified by an instantaneous increase in the population's volume. A flush or unblock procedure at the end of the recorded file almost always resolves the problem. (**A**) “Small” and “large” particle count spontaneously increases. (**B**) “Large” particle count temporarily increases and then decreases, presumably when the aperture is cleared. (**C**) Same as **B** except erroneously measured volume steadily increases through the remainder of the measurement.
(TIF)

Figure S3 **Volume timecourse colormaps before interpolation.** (**A**) Figure 2A (**B**) Figure 2B (**C**) Figure 3B.
(TIF)

Figure S4 **Culture condition comparison.** The modes of volume distributions were recorded from live Multisizer chamber-grown and fixed aliquots of flask-grown parallel cultures used to produce Figure 5. In order to provide a more direct comparison between live and fixed cells, the percent change in volume from the timecourse's start is reported. In elutriated wild type yeast, the culture conditions in a Multisizer sample compartment and a standard flask produce similar results.
(TIF)

Figure S5 **Sample output of MATLAB program provided in Section 2 of Supplementary Documentation (File S1).** (**A**) Volume timecourse of individual pulse data for yeast. Bold horizontal lines represent the bound pairs between which data is used for analysis. Thin green lines are a linear fit of all data across a user-selected moving window. (**B**) Assorted bound pairs for each saved data file in the timecourse. (**C**) Average growth rate for user-selected moving window. Values are calculated from the slope of the linear fits in Figure S5A.
(TIF)

File S1 **Supplementary Documentation** This information provides (1) a line-by-line outline of the critical elements in a Beckman-Coulter Multisizer 4 file, (2) MATLAB code for data extraction and basic analysis, and (3) example output.
(DOC)
