# Supplementary figures and images for: Continuous and Long-Term Volume Measurements with a Commercial Coulter Counter
Source: PLoS One. 2012 Jan 17;7(1):e29866. doi: 10.1371/journal.pone.0029866 (PMC3260162; doi:10.1371/journal.pone.0029866)

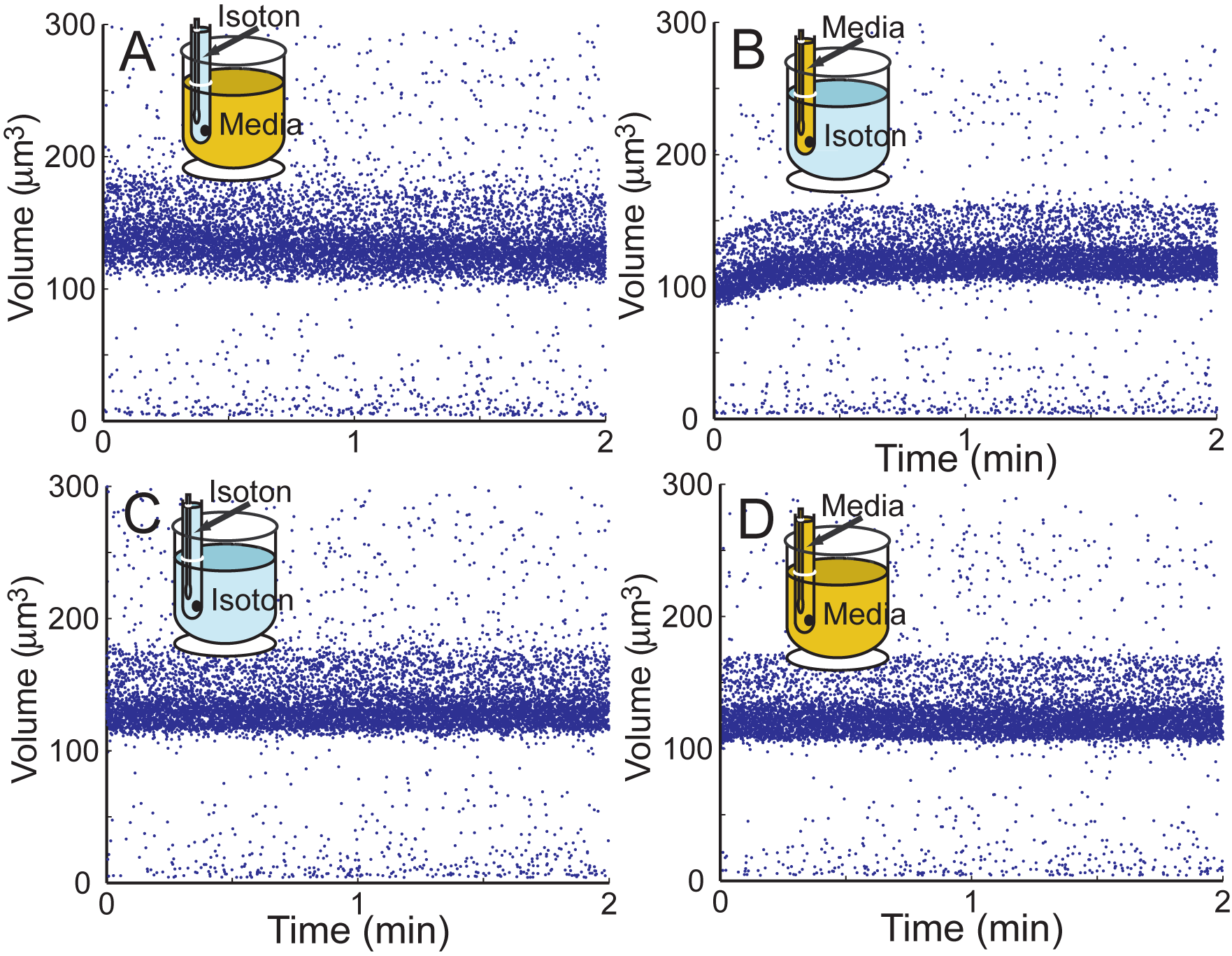

Supplement: Figure S1 — Volume measurements are unreliable (up to 20% error) for mismatched electrolyte and diluent conditions. (A, B) For a mismatched system electrolyte (inside aperture tube) and sample solution (beaker) there is a ∼30 s period required for the measurement to stabilize. During this period the sample solution fills the inside of the aperture tube and finally creates matched solution conditions across the aperture sensing zone. During a continuous measurement, this drift would be observed after every instrument flush, or between each recorded file (every 150 s). (C,D) Volume measurements are stable through the entire measurement if the system electrolyte and sample solution are identical. (TIF) [file pone.0029866.s001.tif]

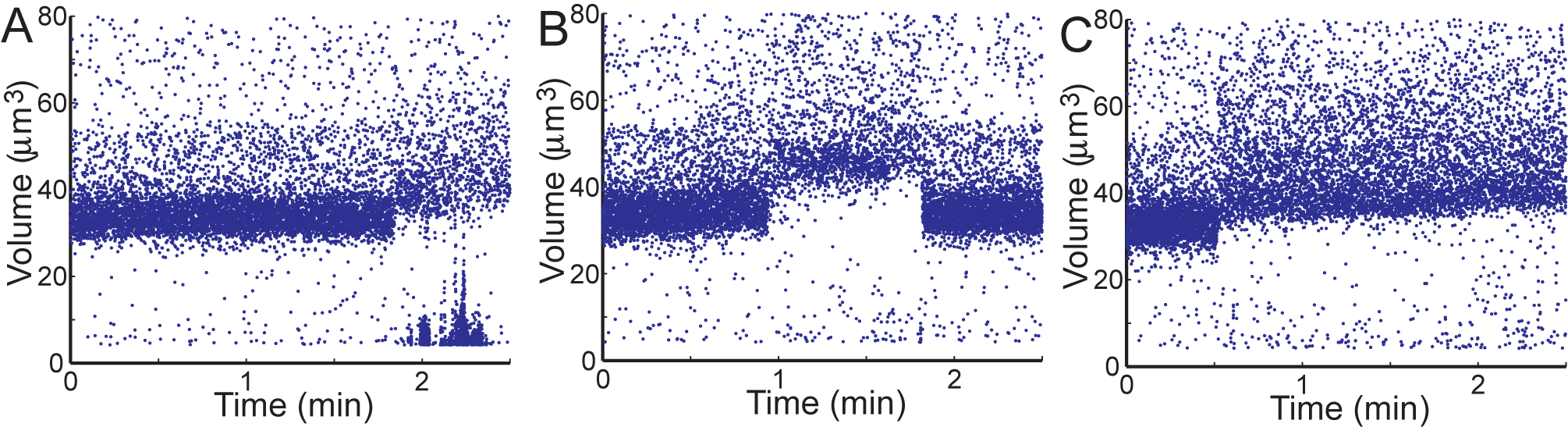

Supplement: Figure S2 — Examples of interference. In general, interference is identified by an instantaneous increase in the population's volume. A flush or unblock procedure at the end of the recorded file almost always resolves the problem. (A) “Small” and “large” particle count spontaneously increases. (B) “Large” particle count temporarily increases and then decreases, presumably when the aperture is cleared. (C) Same as B except erroneously measured volume steadily increases through the remainder of the measurement. (TIF) [file pone.0029866.s002.tif]

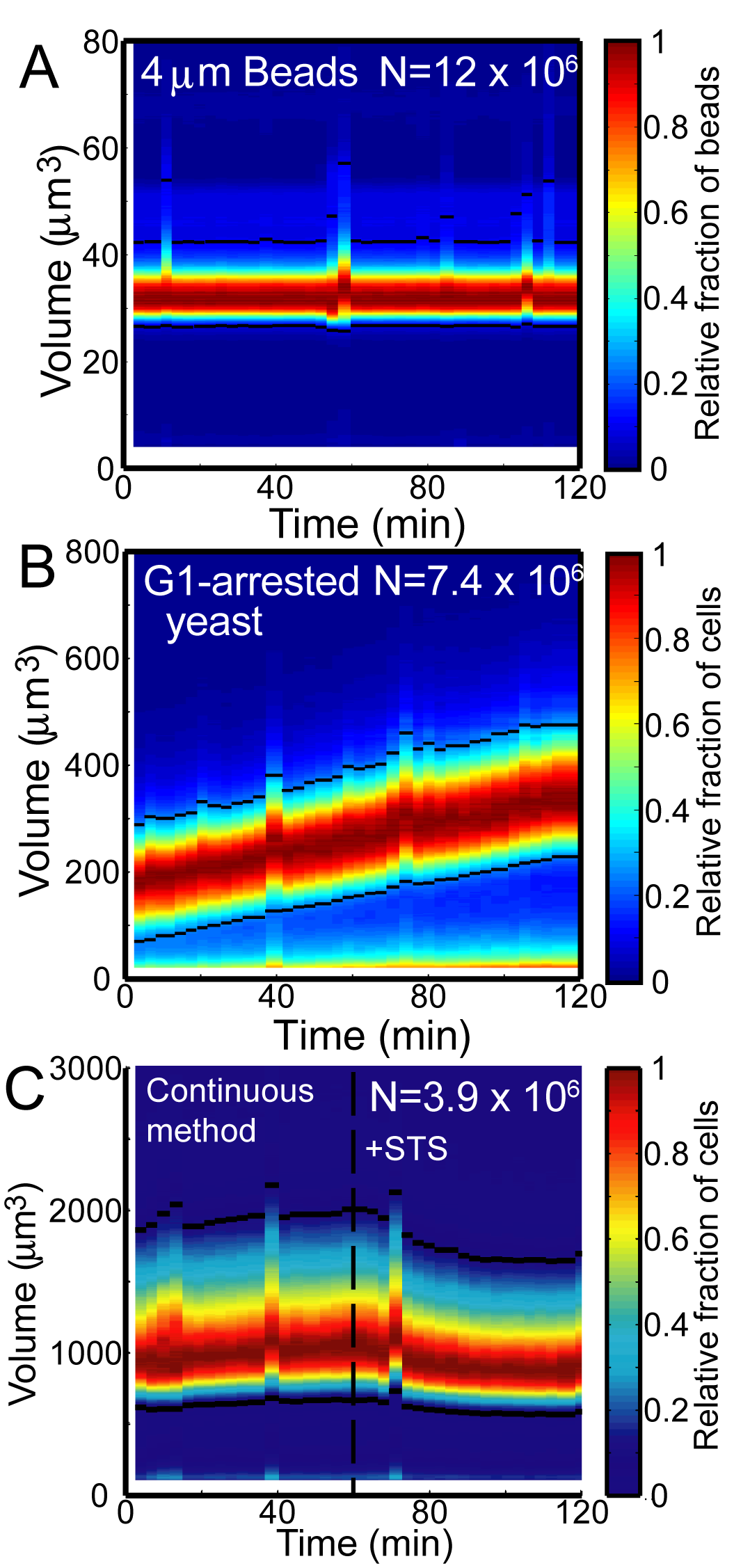

Supplement: Figure S3 — Volume timecourse colormaps before interpolation. (A) Figure 2A (B) Figure 2B (C) Figure 3B. (TIF) [file pone.0029866.s003.tif]

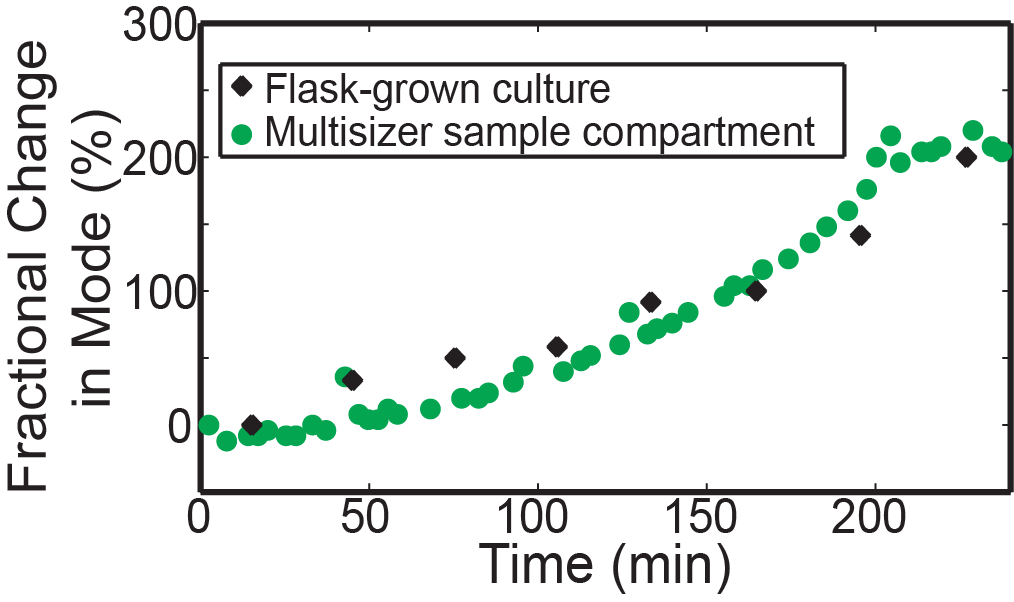

Supplement: Figure S4 — Culture condition comparison. The modes of volume distributions were recorded from live Multisizer chamber-grown and fixed aliquots of flask-grown parallel cultures used to produce Figure 5. In order to provide a more direct comparison between live and fixed cells, the percent change in volume from the timecourse's start is reported. In elutriated wild type yeast, the culture conditions in a Multisizer sample compartment and a standard flask produce similar results. (TIF) [file pone.0029866.s004.tif]

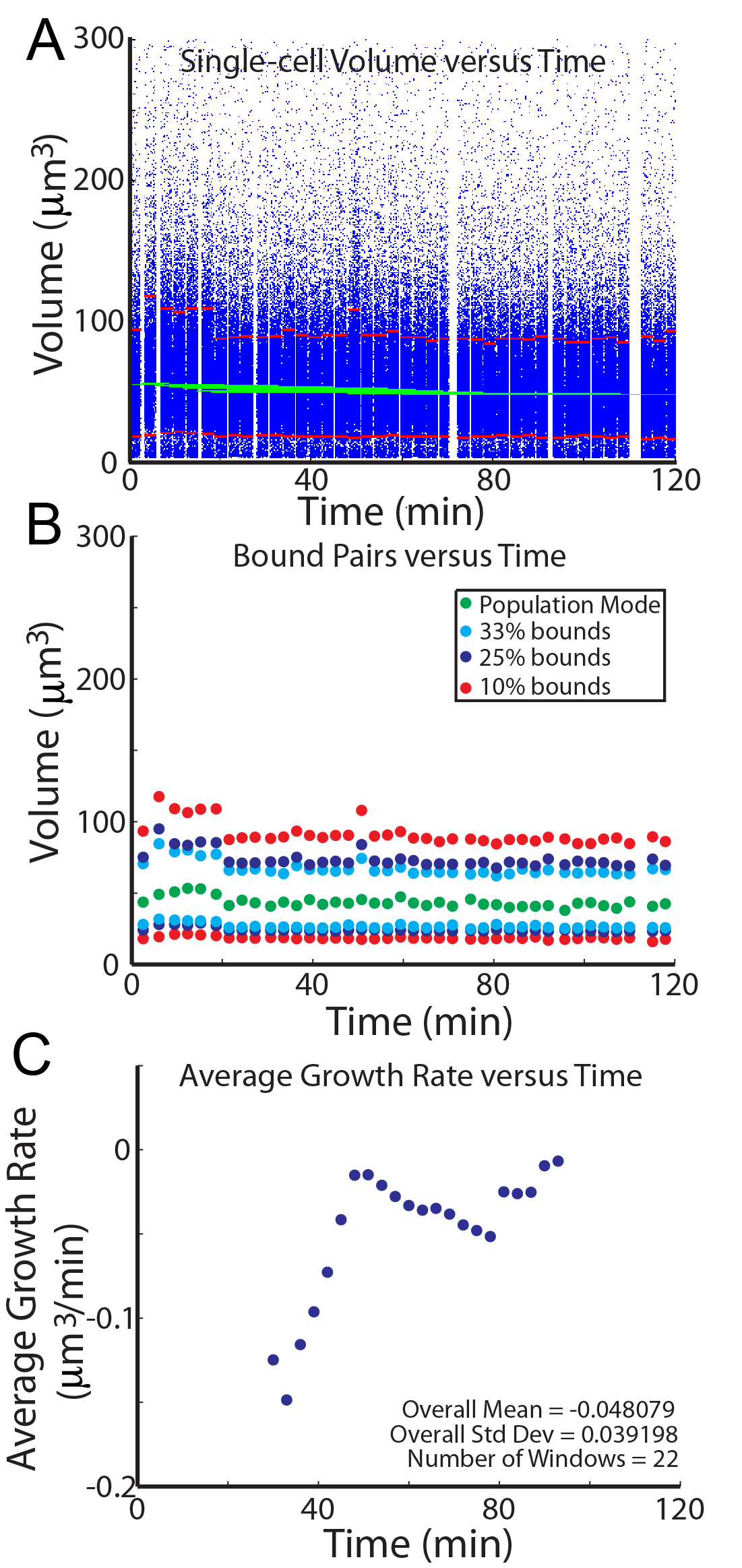

Supplement: Figure S5 — Sample output of MATLAB program provided in Section 2 of Supplementary Documentation (File S1). (A) Volume timecourse of individual pulse data for yeast. Bold horizontal lines represent the bound pairs between which data is used for analysis. Thin green lines are a linear fit of all data across a user-selected moving window. (B) Assorted bound pairs for each saved data file in the timecourse. (C) Average growth rate for user-selected moving window. Values are calculated from the slope of the linear fits in Figure S5A. (TIF) [file pone.0029866.s005.tif]
