# Supplementary material for: Continuous and Long-Term Volume Measurements with a Commercial Coulter Counter
Source: PLoS One. 2012 Jan 17;7(1):e29866. doi: 10.1371/journal.pone.0029866 (PMC3260162; doi:10.1371/journal.pone.0029866)
Supplement: File S1 — Supplementary Documentation This information provides (1) a line-by-line outline of the critical elements in a Beckman-Coulter Multisizer 4 file, (2) MATLAB code for data extraction and basic analysis, and (3) example output. (DOC) [file pone.0029866.s006.doc]

**Supplemental file S1**

This supplementary information provides (1) a line-by-line outline of the critical elements in a Beckman-Coulter Multisizer 4 file, (2) MATLAB code for data extraction and basic analysis, and (3) example output.

**SECTION 1**This sample Beckman-Coulter Multisizer data file provides the user a reference for the data extraction processes of the MATLAB program in Section 2.

*Sample Data File: example.#m4*
Line Number Sample file
…
127 Kd= 127.09
…
135 nPulses= 181321
136 nTimeStamps= 750
…
170 StartTime= 1296235312 12:21:52 28 Jan 2011
…
178 Current= 800
…
184 Gain= 4
…
187 MaxHtCorr= -100
…
199 Time= 150
...

Pulse data for each measured particle is stored as a vector of 5 hexadecimal formatted values that correspond to MaxHeight, MidHeight, Width, Area, and Gain, respectively. The time at which the pulse occurs is calculated from the two sections that follow, TSms and TSpulses.
Line Number Sample file
…
1532 [#Pulses5hex]
1533 EB0,DBC,6C,646,22
1534 21D0,2100,9C,12C9,33
…
182853 CF4,CB4,73,629,22
182854 [#TSms]
182855 240
182856 440
182857 640
…
183602 149860
183603 150000
183604 [#TSpulses]
183605 259
183606 455
183607 754
183608 961
…

**SECTION 2**

This MATLAB program provides complete data extraction from multiple Multisizer 4 files and plots the volume and growth rate timecourses (see Section 3).

% Coulter Counter Reader - Multiple Runs

% Alex Engler

% MIT Biological Engineering, Manalis Lab

%

% This program takes multiple .#m4 files created from a Coulter Counter

% Multisizer 4 and extracts relevant information for analysis.

%

% The program prompts the user for the number of runs (i.e. the number of

% .#m4 files), the time interval within which to average subsets of data,

% and the amount of time to shift by. For example, the defaults of a window

% size of 5 minutes with a frequency of 1 minute would analyze all data

% between 0min and 5min real time, then 1min and 6min, 2min and 7min, and

% so on throughout the timespan of the data. The program reads in relevant

% information using knowledge of the format of the .#m4 file, and proceeds

% to calculate a linear regression for subsets of the volume versus time

% data, with a size and frequency of the regressions determined previously

% by the user. The program will also interpolate points between subsequent

% runs by using data concatenated from both files. The program then plots

% these slopes along with the relevant 95% confidence intervals on time

% versus slope (in um^3/min), as well as the volume versus

% time graph, and the bounds versus time graph.

%

% When graphing multiple files, it can only be used with files that were

% created directly in succession, as it reads the timestamp from within the

% file. When selecting a folder, make sure the folder contains only .#m4

% files and the files are in chronological order.

%

% This version of the program is optimized for averaging slope values that

% are longer than the length of the file.

%% Get user information about runs and analysis, acquire files

clear all % initializes the program by clearing everything

close all % closes all figures

fclose('all'); % closes any open files

clc % clears the command window

% prompt information about how to analyze the data

intavg = input('Enter the time interval for averaging slope (min) [5]: ');

if isempty(intavg)

intavg = 5; % default is a 5 minute

end

intavg = intavg * 60; % convert to seconds for ease

intshift = input('Enter the frequency of data points (min) [1]: ');

if isempty(intshift)

intshift = 1; % default is shifting by 1 minute

end

intshift = intshift * 60; % convert to seconds for ease

% determine the method of acquiring files

disp('Do you want to select an entire folder or pick individual files?')

seltype = input(' Enter 1 for folder, 2 for files [1]: ');

if isempty(seltype)

seltype = 1;

end

if seltype == 2 % if selecting individual files

nRuns = input('Enter the number of files [1]: ');

if isempty(nRuns)

nRuns = 1; % default is 1 file

end

fid = zeros(1,nRuns); % preallocate memory for file identifiers

% open the files

for i = 1:nRuns

[fname,pname] = uigetfile('*.#m4',['SELECT FILE ' num2str(i) '/'...

num2str(nRuns)]); % selects file number i/nRuns

if fname == 0 % if no file selected

disp('Program cancelled');

fclose('all'); % close all open programs

return % end program

end

cd(pname); % changes the current directory to the one with the file

fid(i) = fopen(fname); %opens file for reading

if fid(i) == -1 % if file can't be opened

disp('File could not be opened');

return % end program

end

end

else % if selecting a folder of files

% first, you have to find the folder

folder = uigetdir;

if folder == 0 % if no folder selected

disp('Program cancelled');

fclose('all'); % close all open programs

return % end program

end

% get the names of all files. dirListing is a struct array.

dirListing = dir(folder);

fid = zeros(1,length(dirListing)); % preallocate memory for fid

fidkeep = zeros(1,length(dirListing)); % index for .#m4 files

% loop through the files and open. dir also lists the directories, so

for d = 1:length(dirListing) % have to check for them.

if ~dirListing(d).isdir

fname = fullfile(folder,dirListing(d).name); % use full path

fid(d) = fopen(fname); % open your file here

if fid(d) == -1 % if file can't be opened

disp('File could not be opened');

return % end program

end

if length(fname) > 4

if strcmp(fname(length(fname)-3:length(fname)),'.#m4')

fidkeep(d) = 1; % if it's an .#m4 file, mark true

else

fidkeep(d) = 0; % otherwise mark false

end

else

fidkeep(d) = 0; % if filename shorter than 4, not correct

end

end

end

fid(fidkeep == 0) = []; % gets rid of non-.#m4 files

nRuns = length(fid);

end % selecting files

%% Calculate specs about the length of the run, and preallocate variables

% This section and the next section make use of the structured format of

% the Coulter Counter files. It will jump to specific lines and grab the

% relevant numbers, skipping over a known number of characters to get to

% the relevant information.

% i = window # j = run #

% get start times, end times, and number of pulses for each run

starttime = zeros(1,nRuns); % preallocate

runtime = zeros(1,nRuns);

endtime = zeros(1,nRuns);

nPulses = zeros(1,nRuns);

for j = 1:nRuns

% get the start time of the run

linenum = 170; % Line pointer to the line that has the finish time

astarttime = textscan(fid(j), '%*11s %*n %n %n %n', 1, ...

'delimiter', ':', 'headerlines', linenum-1);

starttime(j) = astarttime{3} + 60*astarttime{2} + 3600*astarttime{1};

fseek(fid(j), 0, 'bof'); % resets pointer to the beginning of file

% get the length of the run

linenum = 199;

aruntime = textscan(fid(j), '%*6s %n', 1, 'delimiter',...

'\n', 'headerlines', linenum-1);

runtime(j) = aruntime{1};

fseek(fid(j), 0, 'bof'); % resets pointer to bof

% get the end time of the run

endtime(j) = starttime(j) + runtime(j);

% get the number of pulses

linenum = 135;

aPulses = textscan(fid(j), '%*9s %n', 1, 'delimiter',...

'\n', 'headerlines', linenum-1);

nPulses(j) = aPulses{1};

fseek(fid(j), 0, 'bof'); % resets pointer to bof

end

% scale the start and end times appropriately so the runs start at time 0

endtime = endtime - starttime(1);

starttime = starttime - starttime(1);

% get the total time and total number of pulses for all the runs;

totaltime = endtime(nRuns);

totalpulses = sum(nPulses);

% calculate the number of averaging windows needed for the run

nWindows = ceil((totaltime - intavg)/intshift + 1);

% nWindows = 1;

% if the averaging window is bigger than the runtime, set to 1 window

if nWindows < 0

nWindows = 1;

end

% assign boundary values for each window

boundary = zeros(nWindows, 2); % preallocate

for i = 1:nWindows

boundary(i,1) = (i-1)*intshift;

boundary(i,2) = boundary(i,1) + intavg;

end

% calculate the max number of files that need to have data loaded in order

% to produce a point on the slope graph

nOpen = ceil(intavg/min(runtime))+1;

% preallocate data variables

xquiet = zeros(max(nPulses),nOpen); % for parsed-down timestamp variable

yquiet = zeros(max(nPulses),nOpen); % for parsed-down volume variable

% preallocate global variables for slope and stddev graphs

time = zeros(1,nWindows);

slope = zeros(1,nWindows);

intercept = zeros(1,nWindows);

lerror = zeros(1,nWindows);

uerror = zeros(1,nWindows);

stddev = zeros(1,nWindows);

pulsecount = zeros(1,nWindows);

% preallocate global variables for overall bounds and peak tracking graphs

maxhist = zeros(1,nRuns);

lbound10 = zeros(1,nRuns);

ubound10 = zeros(1,nRuns);

lbound25 = zeros(1,nRuns);

ubound25 = zeros(1,nRuns);

lbound33 = zeros(1,nRuns);

ubound33 = zeros(1,nRuns);

lbound50 = zeros(1,nRuns);

ubound50 = zeros(1,nRuns);

whichbound = zeros(1,nRuns);

toolow = zeros(1,nRuns);

peakloc = zeros(1,nRuns);

peakht = zeros(1,nRuns);

% declare constants

countspervolt = 1/(4*298.02e-9);

ignorepart = 0.02; % percent of low noise to ignore, increased if needed

firstbound = 1; % flag for checking validity of first data set

hbinsize = 1000; % number of bins

% initialize counters

j = 0; % latest run # being analyzed

k = 0; % # of data files stored in memory

%% Read and analyze data

for i = 1:nWindows % for each window

% if the next file lies within the upper boundary of this window,

% import it, grab the relevant data, perform histogram analysis to find

% the relevant subsection of data, plot it on the real time axes if the

% user is interested, and store the parsed down pulse and time data.

% once all files have been read, ignore.

while (j ~= nRuns) && (starttime(j+1) <= boundary(i,2))

% increment counters

j = j+1; % mark that the next file is being imported

k = k+1; % mark that one more data set is being stored

% get the following data from file j: Kd, nTimestamps, current,

% resistance, MaxHtCorr, pulse data, timestamp data, TSms, TSpulses

% get the Kd value

linenum = 127;

aKd = textscan(fid(j), '%*4s %n', 1, 'delimiter',...

'\n', 'headerlines', linenum-1);

Kd = aKd{1}; % converts the cell array into an integer

fseek(fid(j), 0, 'bof'); % resets pointer to the beginning of file

% get the nTimestamps value

linenum = 136;

aTimeStamps = textscan(fid(j), '%*13s %n', 1, 'delimiter',...

'\n', 'headerlines', linenum-1);

nTimeStamps = aTimeStamps{1};

fseek(fid(j), 0, 'bof'); % resets pointer to the beginning of file

% get the aperture current (mA)

linenum = 178;

aCurrent = textscan(fid(j), '%*9s %n', 1, 'delimiter',...

'\n', 'headerlines', linenum-1);

current = aCurrent{1}/1000; % gets current as an integer in mA

fseek(fid(j), 0, 'bof'); % resets pointer to the beginning of file

% get the gain and convert it to resistance

linenum = 184;

gain = textscan(fid(j), '%*6s %n', 1, 'delimiter',...

'\n', 'headerlines', linenum-1);

resistance = 25*gain{1}; % convert gain to equiv resistance (kohms)

fseek(fid(j), 0, 'bof'); % resets pointer to the beginning of file

% get the MaxHeight Correction

linenum = 187;

MaxHtCorr = textscan(fid(j), '%*11s %n', 1, 'delimiter',...

'\n', 'headerlines', linenum-1);

fseek(fid(j), 0, 'bof'); % resets pointer to the beginning of file

% get the pulse data

linenum = 1533;

pulsearray = textscan(fid(j),'%s %*s %*s %*s %*s', nPulses(j), ...

'delimiter', ',', 'headerlines', linenum-1);

% get the time stamp data

TSms = textscan(fid(j), '%n', nTimeStamps, 'delimiter', '\n',...

'headerlines', 2); % reads the TimeStamp millisecond data

TSpulses = textscan(fid(j), '%n', nTimeStamps, 'delimiter', ...

'\n', 'headerlines', 2); % reads the TimeStamp pulses data

st = fclose(fid(j)); % closes file after getting all useful data

% convert the pulse data to volume

height = (hex2dec(pulsearray{1}) + MaxHtCorr{1})';

diameter = Kd*((height./(countspervolt*resistance*current)).^(1/3));

volume = 4/3*pi*(diameter/2).^3;

% convert the time stamp ms and pulse data to a timestamp vector

TSpprev = 0; % initialize marker

TSmprev = 0; % initialize marker

timestamp = zeros(1,nPulses(j)); % preallocates time vector

for m = 1:nTimeStamps

for n = (TSpprev+1):TSpulses{1}(m)

timestamp(1,n) = (TSmprev + ((TSms{1}(m)-TSmprev)/...

(TSpulses{1}(m)-TSpprev))*(n-TSpprev))/1000;

end

TSpprev = TSpulses{1}(m);

TSmprev = TSms{1}(m);

end

% create a histogram of the data and find the peak of relevant data:

% maximum not in the first 2% of bins (noise). Then calculate 10%, 25%,

% 33%, and 50% bounds of the relevant data

% eliminate the few large outliers to get more accurate bin sizings

ysort = sort(volume);

yrel = ysort(find(ysort>0, round(0.995*nPulses(j))));

% make a histogram and find the maximum frequency not in first 2% (ignorepart).

[freq,xout] = hist(yrel,hbinsize);

lastpart = xout>max(xout)*ignorepart; % logical indexing

maxbin = max(freq(lastpart));

maxloc = find(freq == maxbin);

if length(maxloc) > 1

maxloc = maxloc(1); % if more than one peak, take smaller one

end

% check that the maximum isn't noise. If so, find a subset of data

% that cancels enough noise to find the peak of actual data

while firstbound == 1 && maxloc == 1001-sum(lastpart)

ignorepart = ignorepart + 0.02;

lastpart = xout>max(xout)*ignorepart; % logical indexing

maxbin = max(freq(lastpart));

maxloc = find(freq == maxbin);

if length(maxloc) > 1

maxloc = maxloc(1); % if more than one peak, take small one

end

end

% once the first boundary is found successfully, turn off the flag

firstbound = 0;

if maxloc == 1001-sum(lastpart)

toolow(j) = 1;

end

% initialize bound markers

lo10 = maxloc; % indices for data bin at 10% of the max bin height

hi10 = maxloc;

lo25 = maxloc; % indices for data bin at 25% of the max bin height

hi25 = maxloc;

lo33 = maxloc; % indices for data bin at 33% of the max bin height

hi33 = maxloc;

lo50 = maxloc; % indices for data bin at 50% of the max bin height

hi50 = maxloc;

% calculate 10% bounds

while (freq(lo10) > freq(maxloc)/10) && (lo10 ~= 1)

lo10 = lo10 - 1;

end

while (freq(hi10) > freq(maxloc)/10) && (hi10 ~= hbinsize)

hi10 = hi10 + 1;

end

% calculate 25% bounds

while (freq(lo25) > freq(maxloc)/4) && (lo25 ~= 1)

lo25 = lo25 - 1;

end

while (freq(hi25) > freq(maxloc)/4) && (hi25 ~= hbinsize)

hi25 = hi25 + 1;

end

% calculate 33% bounds

while (freq(lo33) > freq(maxloc)/3) && (lo33 ~= 1)

lo33 = lo33 - 1;

end

while (freq(hi33) > freq(maxloc)/3) && (hi33 ~= hbinsize)

hi33 = hi33 + 1;

end

% calculate 50% bounds

while (freq(lo50) > freq(maxloc)/2) && (lo50 ~= 1)

lo50 = lo50 - 1;

end

while (freq(hi50) > freq(maxloc)/2) && (hi50 ~= hbinsize)

hi50 = hi50 + 1;

end

% set the lower and upper bounds of the data as the bounds, not

% including the noise to the far left

if lo33 == 1

lbound = xout(lo50);

ubound = xout(hi50);

whichbound(j) = 50;

elseif lo25 == 1

lbound = xout(lo33);

ubound = xout(hi33);

whichbound(j) = 33;

elseif lo10 == 1

lbound = xout(lo25);

ubound = xout(hi25);

whichbound(j) = 25;

else

lbound = xout(lo10);

ubound = xout(hi10);

whichbound(j) = 10;

end

% store the bounds data

maxhist(j) = xout(maxloc); % value of max bin height

lbound10(j) = xout(lo10); % 10% bounds

ubound10(j) = xout(hi10);

lbound25(j) = xout(lo25); % 25% bounds

ubound25(j) = xout(hi25);

lbound33(j) = xout(lo33); % 33% bounds

ubound33(j) = xout(hi33);

lbound50(j) = xout(lo50); % 50% bounds

ubound50(j) = xout(hi50);

% scale the x variable appropriately so it represents time elapsed in

% real time rather than time elapsed in the run

timestamp = timestamp + starttime(j);

% graph the pulse data in real time, plotting approximately 10000

% points per run to get an accurate idea of the shape of the data. If

% the bounds are not big enough, do not plot the data.

figure(1)

hold on

divfac = ceil(nPulses(j)/10000); % finds a scaling factor

xreduce = timestamp(1:divfac:length(timestamp)); % ~10000 pts

yreduce = volume(1:divfac:length(volume));

xreduce = xreduce/60; % convert to minutes for graphing

plot(xreduce,yreduce, '.b', 'MarkerSize', 3)

xlabel('Time (min)')

ylabel('Volume (\mum^3)')

title('Volume versus Time')

% eliminate large outliers to make graph more legible

ylim([0 max(ubound10)*2]);

% plot the boundaries. color depends on which bounds are used.

if whichbound(j) == 10

line([min(xreduce);max(xreduce)],[lbound;lbound],...

'LineWidth', 2, 'color', 'r')

line([min(xreduce);max(xreduce)],[ubound;ubound],...

'LineWidth', 2, 'color','r')

elseif whichbound(j) == 25

line([min(xreduce);max(xreduce)],[lbound;lbound],...

'LineWidth', 2, 'color','k')

line([min(xreduce);max(xreduce)],[ubound;ubound],...

'LineWidth', 2, 'color','k')

elseif whichbound(j) == 33

line([min(xreduce);max(xreduce)],[lbound;lbound],...

'LineWidth', 2, 'color','c')

line([min(xreduce);max(xreduce)],[ubound;ubound],...

'LineWidth', 2, 'color','c')

elseif whichbound(j) == 50

line([min(xreduce);max(xreduce)],[lbound;lbound],...

'LineWidth', 2, 'color','m')

line([min(xreduce);max(xreduce)],[ubound;ubound],...

'LineWidth', 2, 'color','m')

end

% parse down the data so the only data carried over is the data between

% the bounds. If the bounds are not big enough, do not save the data

if toolow(j) == 0

inbounds = (volume > lbound) & (volume < ubound);

numquiet = length(find(inbounds));

xquiet(1:numquiet,k) = timestamp(inbounds);

yquiet(1:numquiet,k) = volume(inbounds);

end

end % while (j ~= nRuns) && (starttime(j+1) <= boundary(i,2))

% reshape the data into vectors, eliminating the zero fillers

xquietvec = reshape(xquiet,1,numel(xquiet));

xquietvec(xquietvec == 0) = []; % eliminate the zero elements

yquietvec = reshape(yquiet,1,numel(yquiet));

yquietvec(yquietvec == 0) = [];

% get a time point in the middle of each window

time(i) = boundary(i,1) + intavg/2;

% find slope and stddev data

% first, find the data the falls in window i

smallind = (xquietvec>=boundary(i,1)) & (xquietvec<=boundary(i,2));

xsmall = xquietvec(smallind);

ysmall = yquietvec(smallind);

% if more than 2 points in the window, run a linear regression, get

% the 95% confidence intervals, then grab the pertinent values and

% store them in global variables

if length(xsmall) > 2

% correct x variable to get appropriate intercept

p = polyfit((xsmall - boundary(i,1)), ysmall, 1);

fitresult = fit(xsmall',ysmall','poly1');

conf = confint(fitresult, 0.95);

slope(i) = p(1);

lerror(i) = p(1) - conf(1,1);

uerror(i) = conf(2,1) - p(1);

stddev(i) = std(ysmall);

intercept(i) = p(2);

end

% calculate how many pulses per slope

pulsecount(i) = length(xsmall);

% if the next window does not require the first set of data stored in

% memory, remove it and shift the remaining sets of data over in memory

% to make room for new data when needed. endtime(j-k+1) corresponds to

% the first run data stored in memory. if the last run, ignore.

while (i ~= nWindows) && (endtime(j-k+1) <= boundary(i+1,1))

k = k-1; % mark that one less data set will be stored in memory

for d = 1:k

xquiet(:,d) = xquiet(:,d+1); % shift data left one space

yquiet(:,d) = yquiet(:,d+1);

end

xquiet(:,k+1) = zeros(length(xquiet),1); % clear space for new data

yquiet(:,k+1) = zeros(length(yquiet),1);

end

end

fclose('all'); % closes all open files

% convert all units to minutes

time = time/60; % sec to min

starttime = starttime/60; % sec to min

endtime = endtime/60; % sec to min

slope = slope*60; % um^3/s to um^3/min

lerror = lerror*60; % um^3/s to um^3/min

uerror = uerror*60; % um^3/s to um^3/min

% get rid of all points that are exactly zero. This will be the case if

% there is a window that contains no data.

time(pulsecount == 0) = [];

slope(pulsecount == 0) = [];

intercept(pulsecount == 0) = [];

uerror(pulsecount == 0) = [];

lerror(pulsecount == 0) = [];

stddev(pulsecount == 0) = [];

pulsecount(pulsecount == 0) = [];

% save the endtime data for all runs, including bad ones

endtimeall = endtime;

% get rid of boundary data that uses the 50% bounds because it is very

% noisy

deleterow = toolow == 1;

endtime(deleterow) = [];

maxhist(deleterow) = [];

ubound10(deleterow) = [];

ubound25(deleterow) = [];

ubound33(deleterow) = [];

ubound50(deleterow) = [];

lbound10(deleterow) = [];

lbound25(deleterow) = [];

lbound33(deleterow) = [];

lbound50(deleterow) = [];

peakloc(:,deleterow) = [];

peakht(:,deleterow) = [];

%% Display graphs

% display the average slope versus time graph

figure(2)

clf reset % clears any existing graph data

hold on

errorbar(time,slope,lerror,uerror, '.')

xlabel('Time (min)')

ylabel('Average Growth Rate (\mum^3/min)')

title('Average Growth Rate versus Time')

window(1,:) = xlim; % determines window size in order to write text

window(2,:) = ylim;

% show the overall mean and standard deviation on the graph

str1(1) = {['Overall Mean = ' num2str(mean(slope))]};

str1(2) = {['Overall Std Dev = ' num2str(std(slope))]};

str1(3) = {['Number of Windows = ' num2str(nWindows)]};

text(window(1,2),window(2,2),str1,...

'VerticalAlignment','Cap','HorizontalAlignment','Right')

% display the trendlines on volume versus time graph

figure(1)

for q = 1:length(slope)

line([time(q)-intavg/120;time(q)+intavg/120],...

[intercept(q);intercept(q)+slope(q)*intavg/120],'color','g')

end

if length(ubound10) >= 5 % resize window as needed for data set

uboundsort = sort(ubound10);

%ylim([0 uboundsort(length(uboundsort)-4)*2]);

end

% display the bounds versus time graph

figure(3)

clf reset % clears any existing graph data

hold on

plot(endtime,maxhist,'.g')

plot(endtime,ubound33,'.c')

plot(endtime,ubound25,'.b')

plot(endtime,ubound10,'.r')

plot(endtime,lbound10,'.r')

plot(endtime,lbound25,'.b')

plot(endtime,lbound33,'.c')

xlabel('Time (min)')

ylabel('Volume (\mum^3)')

title('Overall Bounds of Histogram versus Time')

legend('Population Mode', '33% bounds', '25% bounds',...

'10% bounds','Location','BestOutside')

% if only one run was selected, provide a histogram of the data

if nRuns == 1

figure(4)

clf reset

hold on

[freqUF,xout] = hist(yrel,hbinsize);

freq = sgolayfilt(freqUF,3,31); %filter freq data for smoothing

bar(xout,freq)

xlabel('Volume (\mum^3)')

ylabel('Frequency')

h = findobj(gca,'Type','patch');

set(h,'FaceColor','b','EdgeColor','b')

window(1,:) = xlim;

window(2,:) = ylim;

line([maxhist;maxhist],[0;max(freq)],'color','g','linestyle', '--')

line([ubound33;ubound33],[0;max(freq)],'color','c','linestyle', '--')

line([ubound25;ubound25],[0;max(freq)],'color','k','linestyle', '--')

line([ubound10;ubound10],[0;max(freq)],'color','r','linestyle', '--')

line([lbound10;lbound10],[0;max(freq)],'color','r','linestyle', '--')

line([lbound25;lbound25],[0;max(freq)],'color','k','linestyle', '--')

line([lbound33;lbound33],[0;max(freq)],'color','c','linestyle', '--')

end

%%

disp(' ')

disp('Program Complete')

disp(' ')

**SECTION 3**Sample output of the MATLAB program is provided as Supplementary Figure 5.
